# Relationships among Lifestyle Awareness, Age, and Lifestyle-related Diseases in Healthy Japanese Community Residents

**DOI:** 10.31372/20200502.1092

**Published:** 2020

**Authors:** Masayo Nagai

**Affiliations:** AINO University, Ibaraki, Japan

**Keywords:** attitude to health, health awareness, health behavior, health promotion, lifestyle

## Abstract

**Purpose:** It is widely known that the risk of lifestyle-related diseases can be reduced by reviewing lifestyles, and a variety of efforts for their prevention, such as health education, are being implemented. This study examined community residents’ lifestyle awareness, examining their views on their health and lifestyles, age, and lifestyle-related diseases.

**Methods:** Study subjects were 180 healthy people (28 men and 152 women) who participated in a health checkup. Participants answered a questionnaire about their awareness of health and lifestyle and their views of disorders. Subsequent measurements of speed of sound (SOS), acceleration plethysmography (APG), and visceral fat area (VFA) were also obtained.

**Results:** The results of the study suggest that age was correlated with some health-related attitudes and behaviors. When health awareness among members of a group is high, it is necessary to provide them with the required information and continuing intervention to motivate them to continue their health improvement.

**Conclusion:** It seems that health awareness influences lifestyle, and its improvement slows the progress of lifestyle-related diseases and reduces the effects of aging.

## Introduction

It is widely known that the risk of lifestyle-related diseases can be reduced by reviewing lifestyles, and a variety of efforts for their prevention, such as health education, are being implemented (King, Grunseit, O’Hara, & Bauman, 2013; Nagai, Uyama, & Kaji, 2013; Nanri et al., 2012; White, Lenz, & Smith, 2013). Those studies involving healthy community residents examined the relationships among the dietary habits of people, their bone density, arteriosclerosis, and the cross-sectional area of visceral fat. The results suggested that eating styles, rather than the intake of specific food, are more closely associated with the parameters, or indices, of lifestyle-related diseases. Community residents may expect improvement in a few aspects of their lifestyles to significantly prevent diseases. However, if a person focuses on only part of his/her lifestyle and fails to view it comprehensively, the improvements may not be effective. Furthermore, the risk of lifestyle-related diseases may increase over time. Therefore, people with an assessment score higher than the criterion may have little or no risk, and they may have a risk even when the score is lower than the criterion. It is important for community residents who have undergone assessments to review their health and lifestyles, regardless of the results (Nagai et al., 2013; Nagai, Uyama, & Kaji, 2015). It is necessary for health care professionals to understand how community residents view their own lifestyles to provide intervention on a continuing basis. Japanese people’s general health awareness is high, and more than 80% of them consider that they are in good health, according to previous surveys on health awareness (Kondo, 1999). Most community residents become actively involved in health activities. Previous studies suggested that health awareness among people who have not undergone health examinations is lower compared to people who have taken them, their attitudes are more negative, and the rate of people who developed healthy habits is lower (Fuchino, 2002). However, another study suggested that knowledge of healthy lifestyles does not necessarily reduce unhealthy habits (Nanri et al., 2012). Moreover, few studies have examined relationships among health awareness in members of community groups and parameters of lifestyle-related diseases when their awareness is high.

The purpose of this study was to confirm the relationship between age, health perceptions, and lifestyle related disease parameters. We examined the views of healthy community residents on their health and lifestyles, and their relationships with speed of sound (SOS), acceleration plethysmography (APG), and visceral fat area (VFA) scores*—indices* of lifestyle-related diseases. Results may help improve their awareness in general, including dietary habits and physical activities (Nagai et al., 2013) and effectively motivate them to prevent lifestyle-related diseases.

## Methods

### Participants

Study participants included 180 healthy people (28 men and 152 women) (Table 1) who participated in a health checkup between 2004 and 2005. Participants answered a questionnaire about their awareness of their health and lifestyle and their views of disorders that consisted of 15 items. Scoring ranged from the lowest grade as 1 to the highest as 4 (Table 2).

**Table 1:** Mean and Range of Each Parameter in Healthy Subjects

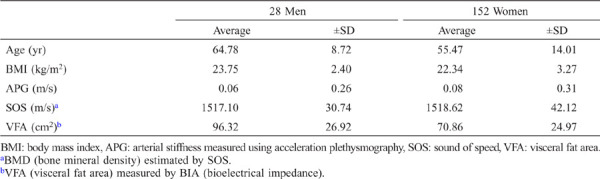

**Table 2:** Items on the Questionnaire

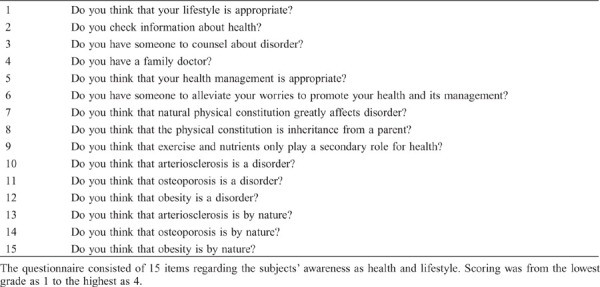

The Institutional Review Board approved and monitored this study. Each participant provided written informed consent.

### Measurements

To evaluate bone quality, speed of sound (SOS) was measured using quantitative ultrasound (QUS, CM-100; Furuno Electric Co. Ltd., Japan) in a calcaneus region. Although the gold standard method to evaluate BMD is dual-energy X-ray absorptiometry (DXA) (van den Bergh, Smals, Schweitzer, & Hermus, 2001), SOS is noninvasive, radiation free, and less expensive than DXA (Della Martina et al., 2008; Tauchmanova et al., 2004). SOS was measured by CM-100, which significantly correlated with BMD measured using DX-2000 and XR-26 (You, 1997).

Arterial stiffness was evaluated using acceleration plethysmography (APG, Artett; U-Medica Inc., Japan). Each subject rested in a sitting position for about 5 minutes before the APG was measured at the second fingertip of the right hand. The APG consisted of four systolic waves; a-, b-, c-, and d-waves (Aiba et al., 1999). The a-peak was set to 100% standard and the b-, c-, and d-peaks were determined by standard on the machine (Aiba et al., 1999). The a-peak was always positive and the b-peak was always negative. The c- and d-peaks may be positive or negative. A positive wave was defined as one above the base line, and a negative wave as one below the base line (Aiba et al., 1999; Elgendi, 2012). Quantitative analyses of the waves were conducted in terms of amplitude ratio (the amplitude of the b, c, or d wave divided by the amplitude of the wave) (Aiba et al., 1999). The amplitude ratios were defined as parameters—b/a, c/a, and d/a (Aiba et al., 1999). In this study, waveform index1 was used as APG that was calculated from d/a-b/a. Waveform index1 reflects peripheral vascular resistance and blood vessel elasticity. The APG wave pattern changes in an age-dependent manner. Therefore, the wave pattern was a fixed quantity and a standard was made. In this study, the standard used was the waveform index 1, indicating the degree of vascular aging. A previous study (Nagai et al., 2013) reported that APG was significantly correlated with baPWV (brachial-ankle pulse wave velocity). Therefore, the present study used APG instead of baPWV to analyze.

The VFA was measured using bioelectrical impedance (BIA, DF515, Yamato-Scale Co. Ltd., Japan). BIA is an accurate, noninvasive method for measuring body composition, especially aqueous components in humans, and for calculating VFA by using inputted information of age, sex, height, weight, and waist circumference. Previous studies have shown that BIA values are well correlated with values measured using computed tomography (CT) (Nagai et al., 2008; Sun et al., 2005). While these methods have limited potential for accurately measuring visceral fat deposition in a clinical setting (Bastard et al., 2006), BIA and anthropometric methods can be useful in classifying adipose tissue distribution for the initial diagnosis of abdominal obesity for individuals, and for general application in epidemiological studies (Bastard et al., 2006).

### Statistical Analysis

Statistical significance was assessed using multiple regression and Pearson’s correlation coefficient analysis by software (IBM SPSS Statistics 21).

## Results

In this study, the older one was, the more often they thought that health management was more appropriate. Also, the older they were, the less likely they could talk to someone about their concerns and receive health support.

As shown in Table 3, “Do you think that your health management is appropriate?” was positively associated with age (*P* = 0.002), “Do you have someone to alleviate your worries to promote your health and its management?” (*P* = 0.016) and “Do you think that obesity is a disorder?” (*P* = 0.012) was negatively associated with age when assessed using multiple regression analysis.

**Table 3:** Independent Factors Contributing to Age

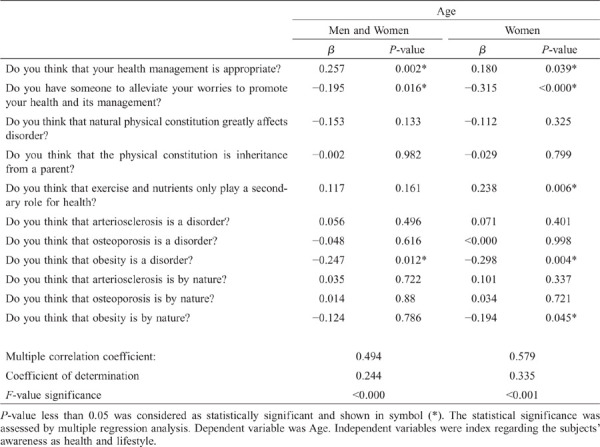

In women, “Do you think that your health management is appropriate?” (*P* = 0.039) and “Do you think that exercise and nutrients only play a secondary role for health?” (*P* = 0.006) was positively associated with age. “Do you have someone to alleviate your worries to promote your health and its management?” (*P* < 0.000), “Do you think that obesity is a disorder?” (*P* = 0.004), and “Do you think that obesity is by nature?” (*P* = 0.045) was negatively associated with age when assessed using multiple regression analysis (Table 3).

The result of analysis using Pearson’s correlation coefficient shows that the SOS was correlated with “Do you think that obesity is a disorder?” (*P* = 0.01, *r* = 0.225) in men and women. Also, VFA was correlated with “Do you think that obesity is by nature?” (*P* = 0.001, *r* = 0.322) in men and women (Table 4).

**Table 4:** Correlation Between Lifestyle Questionnaire Scores and BMI, APG, SOS, and VFA

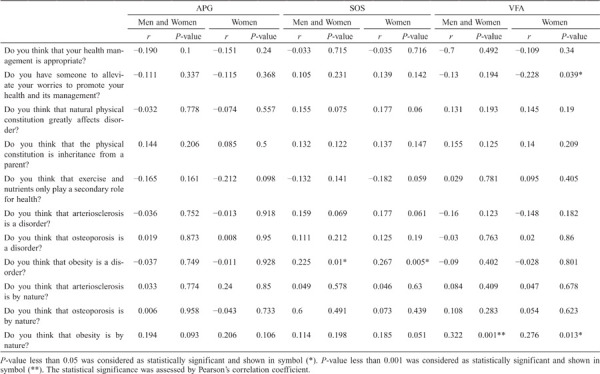

## Discussion

The results of this study suggest that some items regarding the subjects’ awareness of their health and lifestyles were correlated with the age. The older the community residents, the greater the level of satisfaction with their health management. It is known that there are age differences in health behaviors (Table 3). The results of a previous age-specific survey on the lifestyles of Japanese people suggested that the awareness of all lifestyle-related items among people aged 45 or older was higher, or they had developed healthier daily habits, compared to those younger than 45 (Kimura, Endo, & Hiruma, 1999). Another previous study suggested that awareness of the importance of physical activities and dietary habits was high among people aged 65 years or older who attended an exercise class, whereas the awareness of health promotion based on both physical activities and dietary habits was low among people aged between 45 and 64 years old who attended the same exercise class (Oyama & Sakuyama, 2002).

Furthermore, 66% of people in the elderly group (mean age of males and females: 71.7 and 69.1 years old, respectively) stated they always pay attention to the nutritional value of meals, which is higher compared to the young group (mean age of males and females: 20.9 and 18.7 years old, respectively) (15%) (Fukatsu & Sakamoto, 1993). This also suggested that health awareness was higher among elderly people for the following reasons: They had more time to consider it or were more concerned about disorders (Tsushita et al., 1999). In the group of people who independently underwent health checkups in the community, the age was significantly correlated with the level of satisfaction with their health management (Table 3). Similar results were noted in the female group, presumably because the proportion of female subjects was larger in the present study. However, previous studies suggested that health awareness among females was higher compared to males (Fukatsu & Sakamoto, 1993; Nagasawa, Kondou, & Nakajima, 1997) presumably because Japanese females have more opportunities to think about life and health, including the preparation of meals.

Elderly people answered “No” to the question: “Are there any persons around you to alleviate your worries over your health and its management” (Table 3), which suggested that the older the person, the less likely they would receive support from others. According to a previous study, when people received support from others, their QOL was high (Morimoto & Maruyama, 2001).

It is known that psychological health is related to the lifestyle. People need to have someone to advise them or alleviate their worries to promote their own health and QOL (Morimoto & Maruyama, 2001). However, people have fewer acquaintances to share their worries with as they age, although their health awareness and concern over disorders increases. Therefore, the roles of health checkups as social resources are also important. A large number of male subjects stated that they consulted their attending physicians about disorders, whereas more than 40% of the female subjects had no attending physician (Data not shown).

Questions regarding their views of disorders were also asked. The higher the age, the lower the rate of subjects or females who answered “Yes” to the question: “Do you think that obesity is a disorder?” There were a large number of elderly people who stated that obesity is not a disorder.

Osteoporosis is another major health problem and a cause of morbidity and mortality from fracture (Sumino et al., 2007). The bone mineral density (BMD) of Japanese peaks around the age of 25 and is sustained to around age 45, but decreases thereafter (Yanagi, 2003). Postmenopausal women show a rapid decrease in the BMD due to decreased estrogen levels (Schousboe, Nyman, Kane, & Ensrud, 2005), which is a cause of significant gender difference. Arteriosclerosis is a major cause of coronary heart disease and cerebrovascular disease (You, Ryan, & Nicklas, 2004). Advanced arteriosclerosis is associated with age and lifestyle (Nagai et al., 2013), and, therefore, lifestyle improvements are important for prevention. Abdominal obesity as well as arteriosclerosis and osteoporosis are well known diseases and risk factors related to lifestyle (Nakao et al., 2012). Visceral fat accumulation is a cause of insulin resistance, consequently inducing metabolic disorder and cardiovascular disease (You et al., 2004). It is well known that abdominal fat increases with age in males (Nagai et al., 2013) and that it remarkably increases after menopause in females (Kwon et al., 2010). These results reflect the following: arteriosclerosis is associated with cerebrovascular and cardiovascular disorders, osteoporosis is closely related to severe symptoms, such as a bed-ridden status, and the rate of males with obesity is higher. It is necessary to ask participants in health checkups to provide information and implement personal health consultation for them to examine changes in the above-mentioned recognitions and consider what forms of information provision and health consultation are required.

In the results of the present study, the SOS, APG, and VFA scores of community residents were not significantly correlated with their awareness of lifestyles and health. Since people usually undergo health checkups based on their own free will, their health awareness tends to be high, and they are expected to voluntarily become involved in health-promoting activities (Fukatsu & Sakamoto, 1993). However, the results suggest that awareness of healthy lifestyles is not necessarily correlated with health examination measures, or indices of daily habits (Table 4). According to a previous study, people who have developed healthy daily habits tend to give many positive answers to questions related to health awareness (Fuchino, 2002) and, therefore, the recognition of lifestyles actually influences their daily habits. However, they were not significantly correlated with the parameters of lifestyle-related diseases (Table 4), presumably due to the significant influences of the age (Nagai et al., 2013).

Participants in the present study who underwent health checkups also had no lifestyle-related diseases. In the future, it will be necessary to conduct further surveys on a continuing basis to examine changes in their awareness.

It is easier for highly motivated people to improve their daily habits (Kadoma & Shirai, 2002), with the recognition of the results of health checkups helping them become more interested in health management and motivation to improve their lifestyles. It is necessary to understand the problems of individuals and ask about their situations in daily life in order to manage their lifestyles which is an important role of nursing care (Banning, 2005).

Therefore, health checkups serve as an opportunity for examinees to review their lifestyles and promote their own health. When we give health checkups, it is necessary to consider intervention methods according to age.

The results of the present study suggest that the age of people is correlated with some health-related attitudes and behaviors. When health awareness among members of a group is high, it is necessary to provide them with the required information and continuing intervention to motivate them to continue their health improvement. Although health awareness was not correlated with SOS, APG, or VFA scores—indices of lifestyle-related diseases, it was strongly associated with the age in our study. It is clear that health awareness influences the lifestyle, and its improvement slows the progress of lifestyle-related diseases and reduces the effects of aging.

This study found that age was associated with some health consciousness and health behaviors. It was necessary to provide information and motivation to the group for continued high health awareness and suggests that future intervention should be provided.

## Acknowledgments

The study was supported by volunteer participants and nurses.

## Declaration of Conflicting Interests

The author declared no potential conflicts of interest concerning the research, authorship, or publication of this article.
